# Supplementary material for: Are carbon emissions trading and green financial instruments synergistic? -Comprehensive quantitative research based on content analysis
Source: PLoS One. 2024 Mar 7;19(3):e0298601. doi: 10.1371/journal.pone.0298601 (PMC10919601; doi:10.1371/journal.pone.0298601)
Supplement: S1 Data — (ZIP) [file pone.0298601.s001.zip › Data/地级市Z_F工作报告.zip!/╣π╬≈/╟╒╓▌╩╨/└┤╫╘╣1⁄2╓┌║┼í╛┬φ┐╦╩2╛▌═°í┐_2019.docx]

各位代表：

　　现在，我代表市人民政府向大会报告工作，请予审议，并请市政协委员和列席会议的同志提出意见。

　　一、2018年工作回顾

　　过去一年，我们始终坚持以习近平新时代中国特色社会主义思想为指导，认真贯彻党的十九大和十九届二中、三中全会精神，全面贯彻“三大定位”新使命和“五个扎实”新要求，按照党中央、国务院和自治区党委、政府的决策部署，在市委的正确领导下，面对错综复杂的发展环境，我们始终坚持稳中求进工作总基调，坚持新发展理念，落实高质量发展要求，以供给侧结构性改革为主线，持续打好三大攻坚战，统筹稳增长、促改革、调结构、惠民生、防风险，着力做好“六稳”工作，努力构建大开放、大通道、大港口、大产业、大物流发展新格局，全市经济社会各项事业提质增效、平稳发展。据统计，全年地区生产总值增长6%，规模以上工业总产值增长8.6%，固定资产投资增长11.7%，财政收入增长2%，港口货物吞吐量增长21.7%，集装箱吞吐量增长31.3%，社会消费品零售总额增长9.5%，城镇、农村居民人均可支配收入分别增长6.6%和8.6%，城镇登记失业率2.15%。

　　一年来，我们紧紧围绕新时期钦州发展新目标新思路，大力开展“不忘初心再扬帆”解放思想大讨论和优化营商环境“服务企业年”活动，全力推进十大专项重点工作，迎难而上、锐意进取，一些关键领域和重点工作取得了重大突破，五大发展新格局加快推进，“一带一路”西部陆海新通道枢纽城市的集聚态势更加凸显，开放合作之路更加顺畅，高质量发展的条件更加有利，生产要素向钦州加速流动，产业和项目向钦州加快汇集，钦州正以全新的姿态站在新的发展起点上！

　　一年来，我们主要做了以下工作：

　　（一）着力强龙头、补链条、聚集群，支柱产业发展格局实现重大突破。石化产业实现从“一枝独秀”到“三头并进”的重大突破。从中石油广西石化公司单一龙头企业，发展到多个实力雄厚的龙头企业。总投资140亿元的上海华谊一期工业气体岛项目加快建设，总投资150亿元的上海华谊二期丙烯及下游深加工项目加快推进；一期投资350亿元的浙江桐昆芳烃项目落户；总投资102亿元的四川能源投资集团轻烃综合利用项目和总投资800多亿元的新浦化学凝析油综合利用项目签订投资框架协议；总投资200亿元的中石油120万吨石脑油制乙烯项目和总投资196亿元的广西投资集团乙烷制乙烯项目加快前期工作。油、煤、气三头并进格局初步形成。特别自豪的是，我们通过科学谋划，严控石化产业园区用地，依法盘活闲置土地，加快项目用地报批和征收，为龙头企业战略布局留足发展空间，推动上海华谊集团从投资228亿元追加到700多亿元，成为广西最大的单体投资产业项目。同时，通过强强联合，引进了法国苏伊士集团、美国普莱克斯公司、荷兰皇家孚宝集团等世界500强企业。修造船业实现从修船到修造并举的重大突破。中船一期10万吨级修船项目全面投产，2万吨级造船项目加快推进，即将开工建造我国第一艘新规范液化石油气运输船。新能源汽车产业在核心技术和配套上实现重大突破。引进绿传传动系统项目，填补新能源汽车动力总成核心技术空白。恒源电动物流汽车等一批汽车产业配套项目落户，科艺新能源汽车充电桩一期、浦北高迈新能源重型卡车动力电池等项目建成投产。力顺机械公司通过战略合作，加快向新能源汽车升级发展。电子信息产业在强龙头上实现突破。总投资超100亿元的泰嘉超薄玻璃基板深加工项目落户，鑫德利光电二期开工，浦晶光学等项目建成投产，电子信息产业逐渐形成规模。大数据产业在补链条上实现突破。新引进广州讯猫软件有限公司等一批信息服务企业，“必爱旅行网”广西总部大数据与结算中心等平台建成投入运营，基于华为的数字经济生态链加快形成。

　　（二）着力强基础、扩规模、搭架构，通道集聚态势不断凸显。通道流量不断增加。港口货物吞吐量突破1亿吨，顺利实现了亿吨大港梦想。集装箱干线港作用日益增强，吞吐量超过230万标箱。新开通5条外贸集装箱航线，总数达到24条。新开通5条通道班列，全年开行班列1154班、增长20倍，实现与中欧班列无缝衔接。通道功能不断完善。西部陆海新通道5个破瓶颈项目在我市实施，钦州港东站集装箱办理站开工建设，钦州港东航道扩建一期工程加快建设。20万吨级集装箱码头及配套航道前期工作加快推进，钦州保税港区东卡口工程等项目开工建设，钦港铁路与钦大铁路联络线、南钦铁路电气化改造工程等项目建成。钦州船舶交易大厅建成启用。通道架构不断加密。海铁联运班列连接西部六省市，与成都、泸州、宜宾等城市建立战略合作关系，以我市为核心枢纽的通道架构日益密集。

　　（三）着力换思路、抓关键、优服务，财政收入走出连年负增长困局。面对年初预测全年财政收入负增长15%的严峻形势，在加快推进中石油配套基础设施建设和建成一批加油站的基础上，先后三次赴中石油总部，极力说服负责生产经营管理和规划计划等部门理解和支持我们增加税收的迫切愿望，在无法增加全年加工量的情况下，通过调整产品结构增加国内汽柴油销量，优化出口比例增加免抵调库指标，入库税费比年初的预测增加了26亿元，双方还形成了良好的协调沟通机制，为下一步合作发展打下坚实基础；同时多措并举，对重点行业逐一研究制定激励扶持政策，开展精准服务，促进交通运输、建筑、房地产等行业税收较快增长。全年财政收入实现正增长，走出了连年负增长的困境，把看似不可能变成了可能。

　　（四）着力强平台、促合作、抓改革，开放发展取得新成效。平台优势作用进一步发挥，三个国家级平台各项主要经济指标均实现较快增长，在广西北部湾经济区12个产业园区中排名前列。钦州保税港区贸易值增速排名广西北部湾重点园区第一，获批汽车平行进口试点口岸，开展“关税保证保险”、“先验放后检测”等口岸通关创新，全面实现国家标准版“单一窗口”统一申报，口岸核心能力复核验收排名全区第一，全年进口汽车数量同比增长85%，进口酒类突破500万升，“蚂蚁洋货”成为唯一进驻首届中国国际进口博览会的广西品牌；中马钦州产业园区全面实施行政审批承诺制改革，基本实现“园区事园区办”，园区发展进入初步收获阶段，大项目布局实现突破，11个产业项目建成投产，燕窝加工贸易基地一期建成使用；钦州港经济技术开发区综合实力提升，在国家级开发区综合考评中排名全区第一。中国－东盟港口城市合作网络新增6个成员，钦州港与泰国林查班港缔结为国际姐妹港。全市外贸进出口227亿元，利用外资同比增长34.5%，对外直接投资额7.8亿美元、排名全区第一。深入推进供给侧结构性改革，支持企业参与电力直接交易，减少用电成本1.65亿元；为纳税人减免退税45.2亿元；政银企融资信息平台建成运行，光大银行、桂林银行进驻开业，浦北农信联社顺利改制为农商行，我市信用综合排名全区第二。深化“放管服”改革，入驻市民服务中心的政务服务事项中，91.6%实现网上办理，99.1%实现“最多跑一次”、排名全区第一。在全区率先启动“证照分离”等改革，率先设立企业开办、投资项目审批等重要领域综合窗口，办理提速81.7%，各类市场主体同比增长24.4%，全市总体投资环境满意度跃升全区第二。在第15届中国－东盟博览会上，我市签约项目投资总额、重大项目数量、单个项目投资额均排名全区第一。企事业单位公车改革、相对集中行政许可、城市综合执法等改革扎实推进。

　　（五）着力攻脱贫、兴乡村、强县域，城乡发展质量不断提升。完成年度脱贫任务。实现3.8万人脱贫、91个贫困村摘帽。实施脱贫攻坚“百日集中大行动”，筹措财政扶贫资金超过10亿元，发放扶贫小额贷款4.5亿元，村民合作社、产业示范基地实现贫困村全覆盖，集体经济收入3万元以上的贫困村比重达94.4%、排名全区第一。修建20户以上村屯硬化路和砂石路849条，受益贫困村群众50万人。实施乡村振兴战略开局良好。贯彻落实市委《关于实施乡村振兴战略的决定》，开展质量兴农、环境优化等“七大行动”，土地确权基本完成，灵山县率先在全区建成农村产权流转交易市场，农村信用信息系统实现县区全覆盖。全市行政村（社区）集体经济收入全部达标，5个行政村（社区）被授予“‘美丽广西’乡村建设示范村”称号，16个村屯被授予“绿色村屯”称号。新增4个自治区现代特色农业核心示范区，浦北石祖禅茶园获评为中国森林养生基地；广西桂合集团、灵山县宇峰保健食品有限公司获评农业产业化国家重点龙头企业，实现零的突破；电子商务网点覆盖全市85%的行政村（社区），荔枝线上销售占总产量的四分之一；获批创建国家级海洋牧场示范区，钦州渔港经济区列入《全国沿海渔港建设规划》；“钦州大蚝”入选2018中国品牌价值区域品牌前100名排行榜；“钦州辣椒”、“钦北荔枝”通过国家农产品地理标志登记认证保护，“钦州赤禾”、“浦北黄皮”通过审核并公示。县域经济加快发展。灵山八一茶场风电场、浦北龙门风电一期等风电项目和钦南协鑫、通威等光伏发电项目并网发电，钦北肯泰医药等项目开工建设；浦北荣获广西县域科学发展进步县，钦北区获评为全区工业投资评价优秀单位；钦南创建国家全域旅游示范区通过自治区初审，灵山、浦北、钦北列为广西全域旅游示范区创建单位；灵山工业区、浦北县工业集中区列入全区重点县域工业园区。城镇化稳步推进。灵山、浦北加快建设新型城镇化示范县，钦北新城城市功能项目加快建设，灵山陆屋国家级特色小镇和浦北龙门、钦北大寺广西首批特色小镇开展规划编制、产业策划等工作。

　　（六）着力保蓝天、护碧水、守净土，打好污染防治三大攻坚战。面对钦江水质持续恶化的严峻形势，以砸锅卖铁、拆屋当瓦的决心，全力开展查污、截污、拔污、治污、清污，依法取缔禁养区内所有养殖场，清理生猪5万多头、活鸡14万多羽、活鸭9万多羽，基本消除西干渠黑臭水体，钦江水质明显好转。直面中央环保督察“回头看”发现的问题，连夜部署、连夜行动、连续作战，打赢了“散乱污”小冶炼企业污染治理歼灭战，打击非法采砂、海上非法养殖和捕捞取得突破性成效。全市空气质量优良率同比上升4.9个百分点，PM2.5平均浓度同比下降8.6%，南流江、茅岭江等重点流域和近岸海域水环境质量得到改善，钦州资源循环利用基地成功获批为全国50个国家级基地之一。

　　（七）着力打硬仗、解难题、促统筹，重点民生工程加快推进。多年悬而未决、群众怨声载道的市屠宰场搬迁项目如期建成投入使用，西环路、北环路建设瓶颈实现突破，基本打通群众期盼多年的城市环路。港城公交一体化正式运营，安州大道中段、南珠东大街全线贯通，北环大桥等城市主干道建成，河西污水处理厂提标改造工程开工建设并完成主体工程。加强统筹协调，大塘至浦北高速公路等开工建设，南宁伶俐至陆屋二级公路等基本建成，市际交通更加便捷；郁江调水工程复工，钦江饮用水水源取水口上移工程开工建设，新建王岗山水库工程，保障城区用水优质安全；建成滨江东路等工程，白石湖、辣椒槌片区初具规模，滨海新城开发建设框架进一步拉开；建成北部湾大学滨海校区二期工程，北部湾大学历经7年筹建正式挂牌成立；十件为民办实事工程超额完成年度任务，棚户区改造基本建成1.1万套、公共租赁住房分配入住2.7万套，农村危房改造竣工3500多户。

　　（八）着力强保障、促和谐、惠民生，社会事业持续进步。民生保障全面增强。民生支出186.2亿元，占一般公共预算支出比重83.8%。城镇居民最低生活保障标准提高到每人每月620元，农村最低生活保障标准提高到每人每年3860元。实现跨省异地就医即时结算。就业形势总体稳中向好。坚持房子是用来住的、不是用来炒的定位，房价总体平稳。消费潜力进一步释放，汽车、家具等升级类消费分别增长15.8%、38.8%，商品房销售额、旅游总消费分别增长108.4%、39.1%。社会治理全面加强。启动新一轮全国文明城市创建活动，进一步完善“五城联创”机制，集中力量进一步解决了土地闲置、占道经营等一批城市建设管理难题，成功创建首批“广西食品安全示范城市”，率先在全区实现对危险化学品、烟花爆竹等企业安全生产巡查全覆盖，安全生产形势总体平稳。全面开展扫黑除恶专项斗争和禁毒严打整治工作，加强社会治安立体防控体系建设，社会大局和谐稳定，钦南区获评为全国法治县（市、区）创建活动先进单位。科教文体卫事业蓬勃发展。新增11家高新技术企业、3家自治区级工程技术研究中心、2家自治区级企业技术中心。义务教育均衡发展“区检”通过率100%、“国检”通过率75%。中央民族大学附属中学钦州国际学校、市十三中、市十六中等学校招生办学，完成钦南区特殊教育学校迁建，新建、改扩建幼儿园25所、中小学校256所，主城区新增学位4500个。推动优质资源下沉，加快教育和医疗信息化建设，全市完全小学以上学校互联网接入率100%。成功举办纪念改革开放40周年、庆祝自治区成立60周年系列活动，坭兴陶团结和谐纪念瓶获定为自治区回赠中央代表团的礼品。开展纪念冯子材诞辰200周年系列活动，《近代民族英雄》纪念邮票发行，冯子材、刘永福登上“国家名片”。灵山县新圩镇萍塘村入选中国历史文化名村，中山路骑楼街、占鳌巷入选自治区历史文化街区。新增2人入选国家级非遗代表性传承人名单，《窑变千彩》等艺术作品获国家级奖项。世界沙滩排球巡回赛钦州公开赛、第一届钦州坭兴陶文化艺术节暨南向通道陶瓷博览会等国际性、全国性文化体育活动成功举办，钦州蚝情节成为广西文化旅游新兴节庆品牌。持续推进“健康钦州”建设，加大医联体建设力度，家庭医生服务签约覆盖全部建档立卡贫困人口。

　　国防动员、人民防空和海防、民兵预备役、双拥、优抚安置、应急管理等工作进一步加强，军民融合发展不断推进，民族宗教、档案、科协、外事侨务、工商联、新闻出版、广播电视、统计、地方志、供销、地震、气象、水文、哲学社会科学等工作取得新进展，工会、共青团、妇女儿童、残疾人、老龄和关心下一代、红十字等事业取得新进步，海关、海事、消防、税务、金融、供电、烟草、通信、邮政、盐务等为我市经济社会发展作出了新贡献。

　　一年来，我们坚持政治过硬、执行有力的责任担当，持续加强政府自身建设，面对改革发展难题不推诿、不回避，完成了一个又一个急难险重任务。我们坚持依宪履职、依法行政的法治理念，不断加强法治政府建设，自觉接受市人大及其常委会监督和各方监督，人大代表建议和政协提案办结率100%。我们增强务实高效、便民利企的服务意识，进一步转变政府职能，促进企业投资意愿上升、群众满意度提升。我们树立清正廉洁、纪律严明的优良作风，认真履行全面从严治党主体责任，以政治建设统领政府工作，深入推进党风廉政建设和反腐败斗争，贯彻落实中央八项规定及其实施细则精神和国务院“约法三章”，进一步压减“三公”经费，加强审计监督。强化跟踪督查，推动重大决策部署落实。

　　各位代表，事非经过不知难，过去一年取得的成绩，是以习近平同志为核心的党中央坚强领导的结果，是自治区党委、政府正确领导、关心支持的结果，是市委团结带领全市人民努力奋斗的结果，是市人大、市政协和社会各界监督支持的结果。在此，我代表市人民政府，向全市人民，向人大代表、政协委员、港澳台同胞，向各民主党派、工商联、无党派人士和人民团体，向中直区直单位、驻钦部队、武警官兵、消防救援队伍指战员，向所有关心支持钦州发展的同志们、朋友们，致以崇高的敬意和衷心的感谢！

　　在肯定成绩的同时，我们清醒地认识到，我市发展还存在许多深层次矛盾和问题，发展不平衡不充分的问题还比较突出，优势和潜力还没有充分发挥出来。主要体现在：产业结构不合理，新动能支撑不足，经济增长面临下行压力大；可用财力薄弱，财政运行矛盾突出；园区配套基础设施尚待进一步完善，市园统筹、园区协调发展的联动机制还不健全，投资环境有待继续优化；公共服务供给还不能满足群众对美好生活的需要，环境污染问题尚未根治，群众入学难、看病贵等问题依然存在；一些领导干部思想观念不够解放，敢担当的精神和善作为的能力不足，个别党员干部消极腐败现象仍有发生。对于这些困难和问题，我们一定高度重视，采取有力措施加以解决。

　　二、2019年经济社会发展总体要求和主要目标

　　今年是中华人民共和国成立70周年，是全面建成小康社会关键之年，也是我市加快建设“一带一路”西部陆海新通道枢纽城市的重要一年，做好今年工作意义重大。政府工作的总体要求是：坚持以习近平新时代中国特色社会主义思想为指导，全面贯彻落实党的十九大和十九届二中、三中全会精神以及中央、全区经济工作会议精神，落实建设壮美广西、共圆复兴梦想的总目标总要求，持续深入贯彻落实“三大定位”新使命和“五个扎实”新要求，坚持稳中求进工作总基调，坚持新发展理念，坚持以供给侧结构性改革为主线，深入开展“牢记使命启新航”解放思想再讨论、“产业发展攻坚年”、优化营商环境“服务企业提升年”活动，着力攻坚西部陆海新通道建设，着力推动产业高质量发展，着力推动关键领域改革和高水平开放，着力促进城乡协调发展，着力推动生态文明建设，着力抓好脱贫攻坚和民生建设，着力壮大民营经济，保持经济持续健康发展和社会大局稳定，为全面建成小康社会打下决定性基础，以优异成绩庆祝中华人民共和国成立70周年。

　　今年经济社会发展主要预期目标是：地区生产总值增长7%以上，财政收入增长5%，规模以上工业增加值增长10%，固定资产投资增长10%，社会消费品零售总额增长10%，外贸进出口总额增长20%，港口吞吐量增长13%，集装箱吞吐量增长33%，城镇、农村居民人均可支配收入分别增长8%、10%，城镇登记失业率控制在4.5%以内，农村贫困人口减少5.2万。

　　做好今年政府工作，必须把握好以下几个方面：必须牢固树立“四个意识”，坚定“四个自信”，坚决做到“两个维护”，自觉在思想上政治上行动上同以习近平同志为核心的党中央保持高度一致，坚决落实党中央、国务院和自治区党委、政府以及市委的决策部署，确保政府工作始终沿着正确方向前进；必须牢固树立高质量发展理念，深化供给侧结构性改革，大力推进质量变革、效率变革、动力变革，促进经济发展更高质量、更有效率、更加公平、更可持续；必须牢固树立问题意识，有发现问题的敏锐、直面问题的勇气、解决问题的智慧；必须牢固树立以人民为中心的发展理念，在发展中补齐民生短板，全力以赴把群众最关切最期盼的事一件一件解决好，不断满足人民对美好生活的向往！

　　三、2019年工作安排

　　为实现预期目标，今年将重点抓好以下工作：

　　（一）全力推进“三大提升”工程，全面增强核心竞争力

　　第一，要在开放开发格局创造上实现大提升。紧抓西部陆海新通道上升为国家战略的重大机遇，围绕构建“南向、北联、东融、西合”全方位开放格局，实施更加积极主动的开放带动战略，坚持陆海统筹，大力发展向海经济，打造开放升级版的“钦州样板”。主动对接粤港澳大湾区和海南自贸试验区建设，推动与珠江－西江经济带、北部湾城市群城市的合作，以“园中园”方式规划建设承接粤港澳大湾区产业转移园区；加强与西部陆海新通道沿线城市合作，推动与成都、宜宾、泸州等共建川桂国际产能合作产业园进入实质性实施阶段，争取与重庆开展产业战略合作，推动贵阳－钦州“无水港”建设，争取更多通道班列汇聚钦州，探索发展“飞地经济”；继续完善中国－东盟港口城市合作网络，吸引更多东盟港口城市加入；争取开通至欧洲、美洲等远洋航线。加强通道基础设施建设，开工建设钦港支线扩能改造、鹰岭分区车场等项目，推进钦州港东航道扩建一期建成、二期开工建设，建成钦州港东站集装箱办理站、钦州保税港区东卡口工程项目，全面破解海铁联运“最后一公里”瓶颈；开工建设钦州保税港区7－8#自动化集装箱码头，推进港口智能化信息化建设；推进20万吨级集装箱码头及进港航道项目前期工作，加快建设金鼓江5个石化泊位，完成30万吨级进港航道清淤工程，正式运营30万吨级码头及航道，争取新开放码头5个以上，提升通道大进大出能力。提高对外贸易和利用外资水平，服务外向型企业扩增产能，引进一批汽车、红酒等进口企业；深入实施第二轮加工贸易倍增计划，培育壮大加工贸易龙头企业；加大CEPA项目招商，高水平建设钦州保税港区CEPA先行先试示范基地。

　　第二，要在服务企业环境打造上实现大提升。开展优化营商环境“服务企业提升年”活动，扎实推进优化营商环境重点指标百日攻坚行动，紧盯攻坚目标和重点，直面问题，查找差距，严格落实整改任务，力争重点指标达到全国一流水平。不断深化“放管服”改革，推动“一事通办”改革纵深发展，重点深化区域性评估试点、企业投资项目承诺制、“证照分离”、不动产统一登记等改革，持续清理规范行政许可和中介服务事项，打造改革升级版的“钦州高地”。全面落实“云长制”，加快数字政府建设，协同推进广西数字政务一体化平台建设，让数据多跑路、群众少跑腿、企业好办事。深化供给侧结构性改革，在巩固、增强、提升、畅通上下功夫，组织企业申报电力市场交易，降低企业成本；进一步完善政银企融资信息服务平台，扩大政策性融资担保业务，着力破解民营企业融资难、融资贵问题；搭建房屋租赁交易服务平台，优化商品房供应及库存结构，保持房地产市场稳健发展。进一步健全重商、亲商、安商工作机制，出台促进民营经济发展的综合性政策，全面落实放宽公共基础设施、社会事业领域民间投资准入的各类政策措施，建立政企沟通、企业投诉举报处理等平台，全力保障民营企业合法权益，大力支持民营企业转型升级，让钦州成为民营经济发展的热土。

　　第三，要在干事创业氛围营造上实现大提升。牢固树立“担当为要、实干为本、发展为重、奋斗为荣”的为政理念，完善容错纠错机制，为创新者容、为担当者容、为实干者容，激发和保持锐意进取、奋发有为的精神状态，鼓励各级干部解放思想、敢闯敢试，在制度和规矩的范围内放心放手去大胆闯、大胆干，形成敢改革、勇担当、善作为的良好局面。切实规范和改进督查检查考核，把发展任务细化量化到各县区、各部门、各园区，对督查检查项目实行年度计划和审批报备制度，杜绝运动式轮番督查、机械式对照检查。倡导“干多干少不一样、干好干坏不一样”的导向，建立收入与贡献挂钩的园区薪酬新制度，引导优秀年轻干部到产业发展、项目建设一线建功立业、施展才华。

　　（二）奋力实现“十大突破”，全面提升发展支撑力

　　第一，要在市园统筹和园区经济壮大上突破。坚持“市园一体化、园区一盘棋”，健全市园一体化统筹推动机制，集中全市人力、物力、财力支持中马钦州产业园区发展，加快推进电子信息、新能源等重大项目开工建设，实现鑫德利光电二期、科艺新能源汽车充电桩二期、北斗产业园一期等项目建成投产。推进中马钦州产业园区和其他园区协同发展，按照各自优势跨区布局项目，实现中马钦州产业园区与钦州保税港区、钦州港经济技术开发区、钦州高新区、北部湾华侨投资区等联动错位发展，开工建设钦州保税港区国际冷链物流中心、建成奇智纺纱加工扩能等项目，培育引进国际贸易集成商，积极复制推广自贸区改革试点经验，力争获批进境活牛屠宰指定口岸，扩大汽车进口、跨境电商等特色业务。以产业为纽带推进市、县（区）园区统筹发展，促进临港产业链向县域园区广泛延伸，打造临港产业升级版的“钦州模式”。

　　第二，要在重大产业项目推动上突破。在强龙头、补链条、聚集群上下功夫，大力开展“产业发展攻坚年”活动，加大招商引资力度，强化项目要素保障，健全“红黑榜”、考核评价等制度，按照“三强度两指标一率”评价标准对项目（企业）进行综合评价，细化、分解、落实产业发展的具体任务。力争全年新开工建设超亿元产业项目60个，其中，全力推动上海华谊二期丙烯及下游深加工一体化、浙江桐昆芳烃、四川能源投资集团轻烃综合利用、泰嘉超薄玻璃基板深加工、金桂二期高档纸板扩建等5个总投资超百亿元项目，以及恒源电动物流汽车、上海华谊氯碱2个总投资超50亿元项目开工建设；加快上海华谊一期工业气体岛、中船2万吨级造船等65个超亿元在建产业项目建设；力争灵山大怀山风电场、浦北龙门风电二期等14个超亿元项目竣工投产；持续推进新浦化学凝析油综合利用、广西投资集团乙烷制乙烯等签约项目前期工作，争取获得国家批复早日落户。大力发展电子信息产业，全力推动合丰泰集团总部及东莞生产基地搬迁项目落户，开工建设灵山天山电子液晶显示一体化扩建等项目，积极引进光电、液晶模组等项目；大力发展新能源汽车产业，引进战略合作推动力顺轻型载货汽车向新能源汽车转型，加快建设绿传传动系统，开工建设浦北高迈水氢燃料电池项目，竣工投产钦北埃索凯新材料等新能源电池材料项目，推进一批电机、零部件生产等关联配套项目落户；大力推进中石油炼化一体化项目，争取纳入中石油近期优先实施项目清单。

　　第三，要在总部经济培育壮大上突破。出台鼓励总部企业发展办法，研究制订外向型经济、建筑业、现代物流业发展等扶持政策，着力引进交通物流、建筑、房地产、国际贸易等行业总部或子公司落户，引导参与我市开发建设和在我市经营的外地企业在钦设立子公司，鼓励为我市工业企业服务的第三方物流和服务外包企业在我市注册，力争总部经济发展取得明显突破，不断释放经济发展新动能。

　　第四，要在国有平台公司改革重组上突破。按照总体把握、分类处置、逐步实施的原则，以实现公司化、专业化、市场化为方向，加快出台总体方案，细化改革措施，鼓励社会资本、民营资本参与改革重组，健全完善法人治理结构，稳步推进国有平台公司改革重组，加快破解管理体制和治理结构臃肿、经营机制和运营手段粗放、资产结构混乱、债权债务沉重等问题，激发国有平台公司发展活力，实现瘦身、重组、减负、激活、腾飞。

　　第五，要在大物流产业布局上突破。完成物流中长期发展规划，扶持祥龙、伟杰等物流企业壮大发展，大力发展港航服务业，开工建设安通多式联运综合基地等项目，加快建设互联网智能物流中心、公铁联运集散中心、城市物流配送中心和物流企业无车承运人平台，加快建设中国—东盟（钦州宏进）农产品冷链物流园二期、大榄坪综合物流园区、北部湾海铁联运皇马现代综合物流园。构建物流行业龙头，重点引进国内外品牌物流企业，并吸引供应链关联知名企业进驻，带动本地物流环境优化、本地物流企业成长。

　　第六，要在大通道提效降费上突破。从提高效率、提升效益入手，对标先进口岸标准，研究梳理通关效率和港口收费存在的问题，深入实施口岸提效降费行动，加快建设口岸大通关信息平台，推动大通关作业流程再造，建立口岸查验部门与引航、港务等港口管理运营部门业务协同机制，推动国际贸易“单一窗口”功能提升，探索试点推进中新、中马等跨国口岸通关便利化合作，构建智慧通关体系，打造优质高效的大通道口岸通关环境，确保提效降费措施与成效均有突破。

　　第七，要在文化旅游资源开发与整合上突破。创新旅游资源开发体制机制，通过国有平台公司改革重组，加快组建运营市旅游发展平台公司。把三娘湾旅游景区作为旅游产业发展的突破点，引进行业龙头企业开展战略合作，推动三娘湾创建5A级景区。支持钦南创建国家级全域旅游示范区，灵山、浦北、钦北创建广西全域旅游示范区。推进智慧旅游建设，加快旅游与文化、农业、林业等融合发展，盘活千年古陶城，整合资源打造坭兴陶文化旅游产业园，举办第二届中国四大名陶展，大力创建旅游和旅游产品品牌，不断提升钦州旅游的吸引力、影响力。

　　第八，要在以华为为龙头的大数据产业发展上突破。强化资源整合，在钦州高新区数字经济产业园建设大数据产业创业创新中心，在白石湖片区建设大数据企业总部基地，在白石湖兰亭街建成大数据产业展示中心，在市高新技术产业服务中心建设大数据关联企业孵化基地。推动数字钦州建设，启动建设中国－东盟华为云计算及大数据中心一期等项目，加快建设运营钦州华为数字小镇，引进一批云计算、大数据等华为关联企业落户，实现数字小镇产业集聚初具规模，力争我市大数据产业发展走在全区前列。

　　第九，要在进口高端木材加工园区建设上突破。大力发展进口木材加工业，启动进口高端木材加工园区建设，加快完善园区配套设施，着力引进高端木材加工龙头企业，带动一批木材加工企业落户，同时整合符合转型方向的传统木材加工企业入园，加快形成林木深加工产业集群。

　　第十，要在市区城中村改造上突破。全面调查摸底城中村情况，深入推进城镇化低效用地开发，加大闲置土地处置盘活力度，推进城中村污水管网等基础设施建设，重点加快建设上沙村、开工建设沙坪村改造试点，在钦南、钦北各推进一个城中村开展前期工作，积极探索城中村改造开发运作的钦州模式，逐步破解历史遗留难题，开拓盘活土地、激活资源、改善城市面貌的新路子，从根本上提升城市整体功能和品位。

　　（三）加强城市规划建设管理，焕发城市发展新魅力

　　优化城市空间布局。围绕打造“一带一路”西部陆海新通道枢纽城市，加快主城区和滨海新城建设进度，推进临港新城规划建设。进一步理顺滨海新城管理体制，创新滨海新城开发模式，重点推进白石湖片区开发建设，打通白石湖片区与沙井岛片区、茶山江片区城市主干道，加快建设菩提路、嘉兴街等路网及地下综合管廊，实施白石湖水系整治工程，继续完善滨海新城公共设施，推动白石湖片区向茅尾海延伸。支持社会资本参与城市适度连片开发，推进港城一体化，优化港产城融合发展布局，尽快打通北部湾大道至中马钦州产业园区道路，推进钦州至钦州港轨道交通项目，加快中马钦州产业园区城市功能项目建设，促进港口、产业、城市三位一体、互动发展。推进市区至三娘湾高等级公路项目，加快建设市区东部直通海岸的快速大通道，进一步推动城市向海发展。开工建设兰海高速钦州至北海段改扩建工程，推进邕北高铁大塘至吴圩机场联络线和钦州东站至钦州站动车联络线项目加快获批，规划建设钦州经灵山、浦北至玉林高速铁路，加快实现钦州至灵山、浦北、吴圩机场半小时通达，协同推进北部湾经济区同城化、一体化。

　　强化城市建设管理。打造一批便民“暖心工程”，将有限的财力用到群众最需要、最迫切的地方，贯通一批群众关心的小街小巷，疏通拥堵路、打通瓶颈路、修好半边路、建设人行天桥，方便群众出行；完善一批小区小院文化娱乐、体育休闲等便民设施，加快居民小区、学校、公园等场所公共服务设施建设，提高市民幸福指数。打造一批惠民“地网工程”，全面推进地下管网改造，加快河西片区雨污分流改造，完成金海湾东大街、南珠东大街排水管网修复改造，加大投入推进污水管网规划建设，尽快打通污水管网“主动脉”，畅通“毛细血管”。深入开展黑臭水体整治，重点推进黑臭水体整治一、二期工程，基本消除城市黑臭水体。加快建设郁江调水工程，建成钦江饮用水水源取水口上移工程，推进屯六水库引水工程、大马鞍水库扩容工程建设，提高城市供水保障能力。积极发挥城市管理行政综合执法作用，推进城市精细化、网格化管理。推进城市治理市场化，探索环卫保洁市场化运作新模式。稳步推进城市道路和公共场地临时停车收费。支持行业商会组织各类促销展会活动，鼓励社会力量提供教育、文化、健康养老、医疗、家政等服务消费，顺应居民消费提质转型升级新趋势。

　　（四）大力推进乡村振兴，激发城乡发展新活力

　　提升乡村振兴质量。全面实施乡村振兴战略，深入推进农业供给侧结构性改革，保障粮食安全和重要农产品有效供给，突出补齐农业现代化短板和乡村建设短腿。大力发展绿色循环优质高效特色农业，深入实施特色农业示范区增点扩面提质升级工程，推进农产品加工集聚区建设，发展大蚝、荔枝、“海红米”等特色农海产品加工业，打造一批农产品区域公共品牌、企业品牌、产品品牌，创建一批自治区田园综合体，加快建设国家级海洋牧场示范区，争取新增2个自治区级核心示范区，加大农村电商发展力度，推动农村一二三产业融合发展。深化农业农村改革，培育家庭农场等新型农业经营主体，推进农村土地“三权”分置改革和农村集体产权制度改革，全面完成农村土地确权颁证，引导农村土地有序流转。实施数字乡村战略，加快百兆光纤进农村建设，推进“四好农村路”建设，实施一批水库除险加固、灌区节水改造、农村饮水安全等农业农村基础工程。改善农村人居环境，全面启动“幸福乡村”建设，实施农村人居环境整治三年行动、乡村风貌提升三年行动，完成农村垃圾专项治理两年攻坚任务，让农村环境更秀美、农民生活更和美。

　　提升县域发展水平。壮大县域经济，支持灵山陆屋机电产业园一期尽快正式运营，开工建设灵山动漫玩具产业城项目；支持浦北林业循环产业园建设全区重点培育轻工（木业）产业园，向家具制造、交易展示等中高端产业链延伸；支持钦南金窝工业园进口木材加工业加快发展，开工建设钦南风电等项目；支持钦北北部湾中医药健康产业园和林木产业园加快发展，尽快开工建设储力医药、竣工投产锰华新材料等项目。打造市区连接县城的快速通道，尽快开工建设钦州至浦北、钦州港至灵山高速公路项目，加快大塘至浦北至北流高速公路建设。推进城镇化建设，支持灵山“一城四镇”、浦北金浦新区、钦北新城规划建设，进一步拓展县城发展空间。支持灵山陆屋通过全国特色小镇命名，培育建设浦北龙门、钦北大寺等自治区级特色小镇，争取钦南坭兴陶特色小镇列入广西第二批特色小镇培育名录，打造一批小而美、小而强、有产业、有人气的特色小镇。

　　（五）聚焦小康社会底线任务，打赢脱贫攻坚战

　　聚焦目标任务。聚焦“两不愁三保障”，落实打赢脱贫攻坚战三年行动计划，抓好扶贫专项巡视、督查核查发现问题整改，全面加强贫困村基础设施建设、金融扶贫、电商扶贫、控辍保学、危房改造等工作，对标“十一有一低于”、“八有一超”做好各项工作，力争实现71个贫困村摘帽、5.2万贫困人口脱贫，为决胜脱贫攻坚战奠定坚实基础。

　　推进产业扶贫。引导各类资本增加投入，通过龙头企业、农民合作社、贫困村创业致富带头人等经营主体带动特色产业发展，支持贫困村培育“3+1”特色产业，进一步发展壮大村级集体经济。推广“贷牛还牛”、联建联养联种、扶贫车间等新模式，力争实现贫困户产业扶贫全覆盖，进一步激发贫困户内生动力。

　　加强后期扶持。抓好易地扶贫搬迁工作，解决好易地扶贫搬迁群众就业等后续发展问题。加强对已脱贫摘帽村“回头看”，继续关注和扶持已脱贫户，统筹抓好有脱贫任务的非贫困村均衡发展，研究解决收入水平略高于建档立卡贫困户的群体缺乏政策支持等新问题，提高脱贫质量防止返贫，加快健全稳定脱贫长效机制。

　　（六）合力推进绿色发展，巩固提升生态承载力

　　打好污染防治攻坚战。扎实推进生态环境治理，认真开展中央环保督察“回头看”反馈意见、国家海洋督察反馈意见整改工作，着力解决群众反映强烈的环保突出问题，坚决完成整改任务。打好蓝天保卫战，健全联防联控机制，深入开展“散乱污”企业专项整治，推动工业企业烟气达标排放，加强城市防尘抑尘、烟花爆竹禁燃限放管控工作，强化重污染天气预警和应对。打好碧水保卫战，全面落实河长制、湖长制，推进钦江、南流江、茅岭江、大风江、茅尾海和近岸海域水环境综合整治，加强对江砂和海砂出让、开采、运输、销售、使用的管理，严厉打击违法采砂行为，完成县级饮用水水源地环境问题整改，重点开展钦江水质综合治理，推进主城区钦江两岸绿色生态长廊建设，确保钦江水质全面好转、持续稳定。打好净土保卫战，实施土壤污染风险管控与修复治理工程，建设土壤环境质量监测网络，全面加强危险废物规范化管理，加快推进法国苏伊士集团固废处理等项目。

　　加强生态文明建设。开展生态文明教育。健全生态环境管理制度，抓好山水林田湖海生态保护和修复，加快建设国家森林城市。尽快划定生态保护红线、永久基本农田整备区、城镇开发边界，落实最严格耕地保护制度。优化能源结构，加强重点行业节能减排工作。加大生态环保基础设施建设力度，加快完成河东、河西和县城污水处理厂提标改造工程，推动建设三娘湾污水处理项目，加强钦江沿线镇级污水处理厂运行监管，建立镇级污水处理收费体系，探索将镇级污水处理项目由专业企业统一运营管理。

　　加快发展生态经济。树立和践行“绿水青山就是金山银山”的理念，推进绿色工业体系建设，推行水污染、大气污染、固体和危险废物第三方治理，培育一批国家级、自治区级循环经济示范企业、绿色工厂。建设钦州国家级资源循环利用基地，实施一批节能环保、新能源应用、可再生能源开发等生态经济项目。打好绿色牌、长寿牌、富硒牌，推进绿色、有机高品质农产品开发。

　　（七）努力保障改善民生，不断增强群众获得感

　　提高社会保障水平。完善养老、医疗保险制度，继续提高城乡低保标准、残疾人两项补贴和孤儿基本生活最低养育标准，启动城镇困难群众脱困解困工作，做好关爱农村“三留守”人员工作。继续推进棚户区改造、农村危旧房改造等保障性住房建设。实施就业优先战略和更加积极的就业政策，加大职业技能培训力度，加强困难群体就业援助工作，支持农民工和大中专毕业生就业创业，力争城镇新增就业1.5万人，农村劳动力转移就业新增2.3万人。继续开展拖欠农民工工资专项治理，维护劳动者合法权益。开展城乡居民增收专项激励计划试点，积极落实机关事业单位收入分配改革政策，促进城乡居民就业和收入增长。

　　提高社会治理水平。深入推进扫黑除恶专项斗争和禁毒严打整治，严厉打击各类违法犯罪活动。建设更高水平的平安钦州，贯彻落实社会治安综合治理领导责任制，进一步推进镇（街道）综治中心标准化、农村和城乡结合部“雪亮工程”建设，做好矛盾纠纷精准排查和精细化解、社会治安重点地区和治安突出问题整治等工作。加强城乡社区治理创新，引导社会组织健康有序发展。继续推进“五城联创”。开展食品药品等各类安全风险隐患的排查、防控、化解和整治，抓好安全生产各项工作，加强应急保障、突发事件应对处置、防灾救灾和消防救援能力建设，确保安全形势稳定。深入推进道路运输治违治超工作，强化超限超载源头治理。坚持底线思维，强化风险意识，加强政府债务规范管理，积极防范化解金融风险。

　　提高社会事业发展水平。大力实施为民办实事工程，重点做好社保、健康、教育、水利、安居、农补、生态、文化、扶贫、科技等10个方面40多个惠民项目。强化科技创新支撑，力争新增一批高新技术企业。优化提升钦州科教园区规划，支持广西英华国际职业学院升本，筹建钦州幼儿师范高等专科学校，续建、改扩建、新建一批教育项目，加快消除义务教育“大班额”，确保全部县区通过县域义务教育均衡发展国家评估。深化以新高考为龙头的教育综合改革，加快教育信息化建设成果推广应用。全面推进“健康钦州”建设，深化医药卫生体制改革，推动紧密型医联体建设，实施好基本公共卫生服务项目，提高重大疾病防控能力。围绕庆祝中华人民共和国成立70周年，推出精品力作，策划组织开展丰富多彩的群众文化活动，继续办好一系列重大体育赛事活动。持续推进中国历史文化名城创建工作，加大历史文化街区保护力度。加强双拥创建和国防教育，做好国防动员、退役军人服务管理、优抚安置、民兵预备役和人防海防等工作。落实第四次全国经济普查任务。充分发挥工会、共青团、妇联等群团组织的作用，大力实施妇女儿童“两规划”，继续推进民族宗教、档案、外事侨务、打私、统计、地方志、供销、哲学社会科学等事业全面进步。

（八）切实加强自身建设，树牢担当实干廉洁政府形象

　　敢担当重实干。以舍我其谁的使命感、一抓到底的责任感、只争朝夕的紧迫感、奋斗为荣的成就感，落实自治区重大项目建设、产业大招商、优化营商环境三大攻坚突破年活动各项部署，全力开展“产业发展攻坚年”、优化营商环境“服务企业提升年”活动，推行目标清单、任务清单、责任清单、保障清单工作方法，建立重点工作挂图作战、倒排工期、跟踪督查、结果通报机制，确保上级和市委决策部署落地生根、开花结果。

　　推进法治政府建设。严格依法行政，确保政府工作始终在法治轨道内运行。加大普法力度，深入推进政务公开，着力推行政府决策科学化民主化法治化，认真落实市人大及其常委会各项决议决定，提高人大代表建议和政协提案办理质量，自觉接受人大法律和工作监督、政协民主监督、监委监督及社会监督，加强审计监督。依法深化行政管理体制改革，稳步推进生产经营类事业单位等改革，完成市、县（区）机构改革。

　　加强廉洁政府建设。严格落实全面从严治党责任，扎实做好政府系统党风廉政建设和反腐败工作，加强对重点领域、关键环节、重要岗位权力运行的监督制约，持续整治群众身边腐败和作风问题。进一步转变机关作风，深入贯彻落实中央八项规定及其实施细则精神，大力整治形式主义、官僚主义，严惩懒政怠政、失职渎职行为，全力营造风清气正的发展环境。

　　各位代表，宏伟蓝图已绘就，目标任务已明确，让我们更加紧密地团结在以习近平同志为核心的党中央周围，在自治区党委、政府和市委的正确领导下，不畏艰难险阻，勇往直前，奋力拼搏，在建设“一带一路”西部陆海新通道枢纽城市的新征程上，努力创造出无愧于时代的发展成就，开创钦州更加美好的未来，以优异成绩向中华人民共和国成立70周年献礼！
